# A new TaqMan method for the reliable diagnosis of *Ehrlichia* spp. in canine whole blood

**DOI:** 10.1186/s13071-018-2914-5

**Published:** 2018-06-18

**Authors:** Kirsty Thomson, Tal Yaaran, Alex Belshaw, Lucia Curson, Laurence Tisi, Sarah Maurice, Guy Kiddle

**Affiliations:** 1 ERBA Molecular, Bartholomew’s Walk, Cambridgeshire Business Park, Ely, Cambridgeshire, CB7 4EA UK; 2 Biogal, Galed Labs Acs Ltd, 1924000 Kibbutz Galed, Israel

**Keywords:** PCR, PCRun®, Clinical validation, Emerging, Tick-borne

## Abstract

**Background:**

Ehrlichiosis is an important emerging infectious disease of the canid family and humans worldwide. To date, no extensive evaluation or validation of a molecular diagnostic test for ehrlichiosis has been published. Here, we present data for a newly designed TaqMan assay and compare its performance to a commercial technology (PCRun®). Both of these real-time methods of analysis were evaluated using a comprehensive number of prospective and retrospective samples collected from dogs exhibiting symptoms of ehrlichiosis.

**Results:**

Whole blood samples collected from dogs, retrospectively in the United Kingdom and prospectively in Israel, were analysed for the presence of *Ehrlichia canis* and *Ehrlichia minasensis* DNA using the TaqMan PCR, developed specifically for this study. The results were compared to those of a real time commercial isothermal amplification method (PCRun® system developed by Biogal Galed Labs ACS, Galed, Israel). The sensitivity and specificity (CI: 95%) of the TaqMan PCR and PCRun® were both determined to be 100% and absolute, for all of the samples tested. Interestingly, both tests were demonstrated to be highly comparable, irrespective of differences in amplification chemistry or sequences targeted. Host differences, incidence of disease and geographical location of the isolates had little impact on the positivity recorded by each of the diagnostic methods.

**Conclusions:**

It was evident that both amplification methods were equally suited for diagnosing canine ehrlichiosis and while the PCRun® clearly amplified all clinically relevant *Ehrlichia* species known to infect dogs and humans, the TaqMan method was more specific for *E. canis* and *E. minasensis.* This work demonstrates that despite good analytical sensitivities and specificities for *Ehrlichia* spp. neither method could fully account for the clinical diagnosis of thrombocytopenia.

**Electronic supplementary material:**

The online version of this article (10.1186/s13071-018-2914-5) contains supplementary material, which is available to authorized users.

## Background

**Fig. 1:**
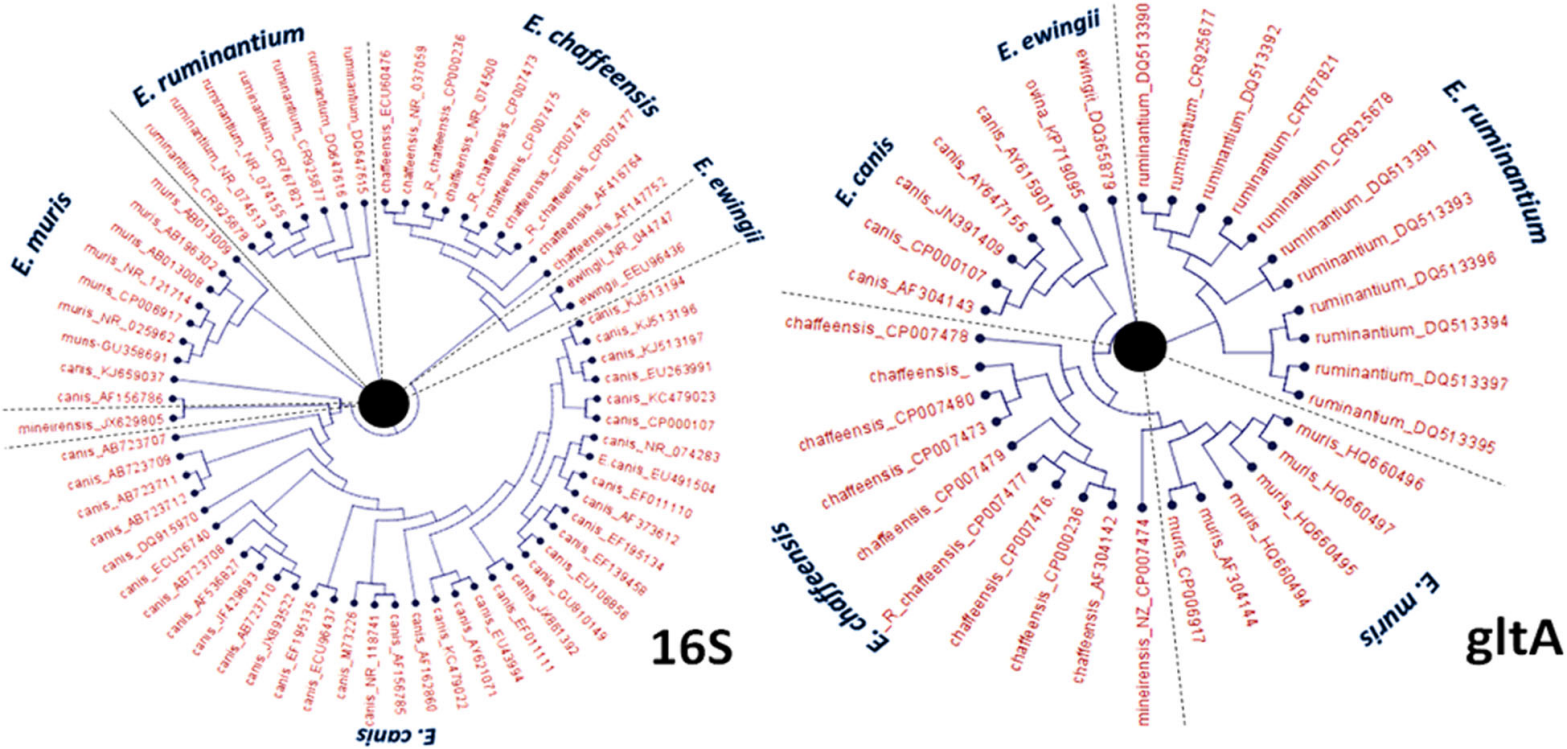
Comparative phylogenetic analysis of 16S rRNA- or gltA-coding sequences from accessions harboured within *Ehrlichia* genomes. GenBank database accession numbers are indicated in the cladogram of all representative species retrieved from GenBank at the time of publication; further information about the accessions used provided in Additional file 1

**Fig. 2:**
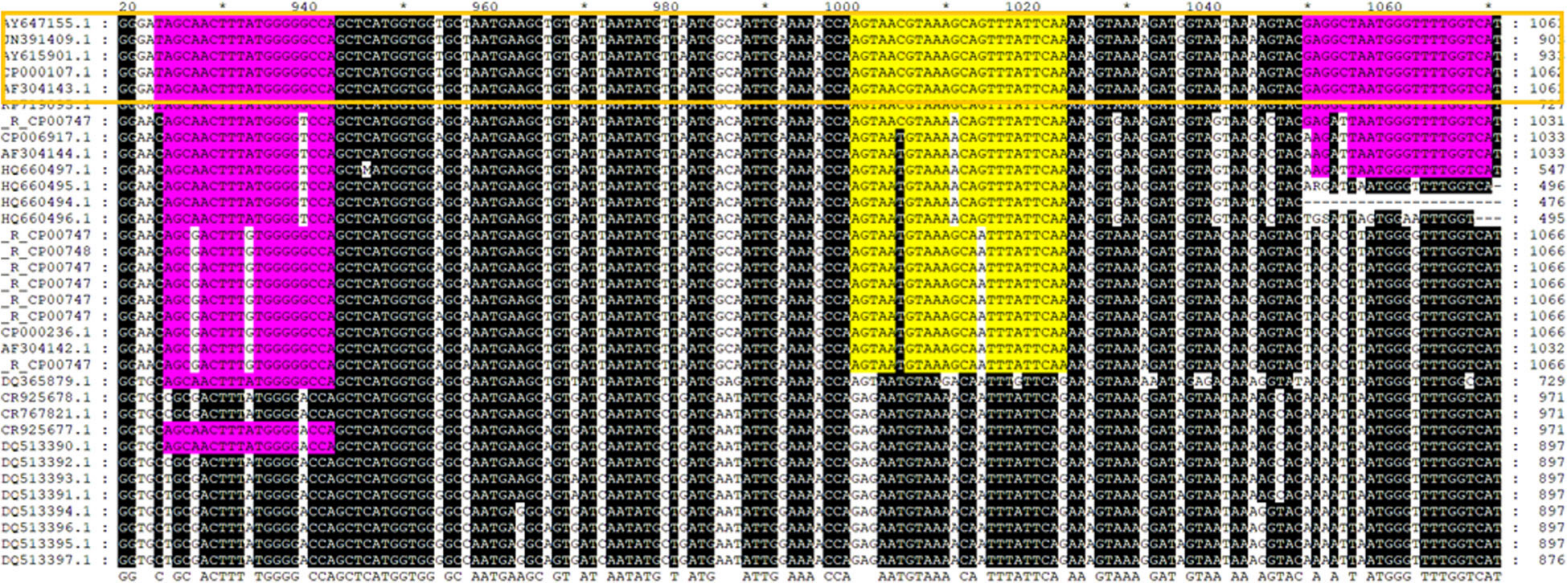
The specificity of the gltA TaqMan primers were validated *in silico* by assessing the primer sequence conservation with respect to the multiple alignment of available target sequences. Orange outlined box identifies *Ehrlichia canis* sequences only

Ehrlichiosis is defined by a number of infectious bacteria, many of which are named according to the predominant host species [1]. Of all the *Ehrlichia* species isolated and characterised to-date *E. canis* and *E. minasensis* are the most closely related, based on phylogenetic analysis of the *16S* ribosomal and *gltA* sequences (Figs. 1 and 2). *Ehrlichia minasensis* was defined as a new *Ehrlichia* species in 2016 after isolating the bacterium from a Brazilian tick (*Rhipicephalus microplus*; [2])*.* This bacterium cultured well in canis DH82 cells [3], despite being isolated from a tick associated with cattle. Ehrlichiosis is considered to be an emerging and life-threatening anthropozoonosis. It is now a notifiable disease and the Centre for Disease Control has reported a steady increase in the prevalence of the human disease cases in the USA. The first case was reported in 1986, and over 900 cases are now reported annually, with at least one fatality each year [4]. Together with tick-borne infections, a number of cases of ehrlichiosis have been contracted via blood transfusions and organ transplantations [5]. With accurate diagnosis and treatment, the prognosis for ehrlichiosis infections is generally good, but too often inappropriate treatment resulting in subclinical infections are reported [6]. The indirect fluorescent-antibody assay (IFA) is still considered to be the diagnostic gold standard protocol [7]. Adverse blood counts, serology or positive IFA tests often lead to a referral for more specialised molecular diagnostics such as PCR, which have the advantage of detecting the organism in the blood [8].

A number of PCR methods have been published over the last two decades, many of which are suitable for end-point detection of *Ehrlichia* infections [9–15]; however only a limited number of these methods have been developed for the real-time detection of *Ehrlichia canis*. The limitations of end-point detection are well documented. The extended analysis time and the possibility of cross-contamination make these methods inappropriate for diagnostic purposes. Real-time PCR assays, which rely upon intercalating dyes report any and all amplifications, whereas probe-based approaches provide greater specificity [16]. Seven previously published PCR primer sets for *E. canis* (Table 1) were assessed for their relevance as a diagnostic tool. Those using end-point or non-specific detection methods were discounted for use as diagnostic tools for Ehrlichiosis [16]. Only two of the published PCR tests were for the real-time probe-based detection of *E. canis*; and these both targeted the *dsb* gene [14, 15]. The sensitivity of one of these primer sets, Michelet et al. [15], was unpublished and analyses of the methods revealed the requirement for a pre-amplification PCR step. The remaining primer set did not perform successfully in our hands [9]. We conclude that no PCR assay published thus far for *Ehrlichia canis* was suitable for diagnosis of canine whole blood samples, and subsequently a new TaqMan assay was developed to target a more divergent gene, citrate synthase.

**Table 1:** The evaluation of published PCR primer designs recommended for the specific amplification and diagnosis of *E. canis*. Comparative data for each published PCR primer design with respect to predicted size and detection method used

Primer name	Target gene	Primer sequence (5′–3′)	Product size (bp)	Detection method	Reference
ECp28-F	*p28*	ATGAATTGCAAAAAAATTCTTATA	843	on-gel	[10]
ECp28-R		TTAGAAGTTAAATCTTCCTCC			
CANIS	*16S*	CAATTATTTATAGCCTCTGGCTATAGGA	413	on-gel	[11]
GA1UR		GAGTTTGCCGGGACTTCTTCT			
16S-fwd	*16S*	TCGCTATTAGATGAGCCTACGT	125	qPCR-SYBR	[14]
16S-rev		GAGTCTGGACCGTATCTCAGT			
EC1	*16S*	TTATAGCCTCTGGCTATAGG	501	Chemiluminescence	[12]
EC2		CACTTTTAACTTACTAGTCC			
DSB-321	*Dsb*	TTGCAAAATGATGTCTGAAGATATGAAACA	350	TaqMan	[9]
DSB-E.canis		AGCTAGTGCTGCTTGGGCAACTTTGAGTGAA			
DSB-671		GCTGCTCCACCAATAAATGTATCYCCTA			
Eh_ca_dsb_F	*Dsb*	AATACTTGGTGAGTCTTCACTCA	110	TaqMan	[15]
Eh_ca_dsb_R		GTTGCTTGTAATGTAGTGCTGC			
Eh_ca_dsb_P		AAGTTGCCCAAGCAGCACTAGCTGTAC			
ECF	*gltA*	CAGGAGTATATGCCTCCTGA	509	on-gel	[13]
ECR		GTTACTTGGTTTTTCAATTGCC			

The TaqMan method described in this manuscript is the first probe-based assay that utilises an alternative genomic target, citrate synthase (*gltA*). Only one previously published primer set for *E. canis* also targeted *gltA* [13]; however, due to the size of the PCR product it was unsuitable for conversion to a real time method. This is a preferable target as it has the most variation between *Ehrlichia* species [17]. Citrate synthase has also been previously targeted to specifically detect a closely related canine pathogen, *Anaplasma platys*, with a high sensitivity [18]. The new TaqMan PCR method was also evaluated on over 200 clinical samples, collected from dogs within the UK and across two regions of Israel. The performance was benchmarked to a recently launched isothermal molecular diagnostic for *Ehrlichia* species (PCRun®). This technology is marketed as an accessible and reliable alternative to PCR testing [19]. The qualitative isothermal amplifications take place in a dedicated reader, where detection takes no longer than 60 minutes. Assays can be performed at either a clinic or laboratory due to the minimal steps involved in the procedure. The dedicated PCRun® reader relays results in real time, which can be confirmed with a secondary device without opening the reaction tubes or a pipetting step.

## Methods

### Samples

Cultured *Ehrlichia canis* was acquired from Professor Shimon Harrus (Koret School of Veterinary Medicine, at the Hebrew University of Jerusalem, Israel). The bacteria were isolated in Israel from a naturally infected dog exhibiting acute signs of the disease and passaged experimentally to a beagle, from which it was propagated in primary canine monocytes, and grown in vitro in a continuous canine cell line, DH82 [20]. *Ehrlichia minasensis* DNA was obtained from the Tick Cell Biobank at The Pirbright Institute and Dr Pilar Alberdi of Instituto de Investigación en Recursos Cinegéticos IREC, Ciudad Real, Spain who were our source for a culture that was maintained according to Cabezas-Cruz et al. [2].

The isolates of *Ehrlichia chaffeensis*, *Ehrlichia ewingii*, *Anaplasma platys*, *Anaplasma phagocytophilum* that were used in the inclusivity study were isolates from infected dog blood that had been previously characterised by PCR at the Vector Borne Disease Diagnostic Laboratory, North Carolina State College of Veterinary Medicine and Biogal [19].

Extracted genomic DNA was quantified via two methods: (i) measuring the sample absorbance between 230 and 300 nm on a NanoDrop spectrophotometer; and (ii) Qubit Fluorometric Quantitation (Thermo Fisher; according to the manufacturers guidelines). DNA integrity was determined by agarose gel electrophoresis; 10 μl of diluted DNA (10–50 ng) was resolved on 1.5% TAE agarose gels (containing a 10^-5^ dilution of Gel Red; Biotium, Fremont, USA) by electrophoresis at 100V for 60 min and visualized by UV fluorescence using an Ingenious Gel Documentation System (Syngene, Cambridge, UK).

### Primers

Upon review of the literature, the citrate synthase (*gltA*) gene was targeted [17, 18]. A conventional method was employed to develop the design. This consisted of an alignment generated by retrieving representative *Ehrlichia* genus target Citrate Synthase (*gltA*) accessions from GenBank using the NCBI BLAST search tool [21]; these retrieved sequences were then aligned using MAFFT [22]. Primer designs were then annealed to the alignment to determine if the sequence targeted was fully inclusive or exclusive for *E. canis.* The multiple alignments and primer conservation were viewed using GeneDoc [23]. All primer sets were screened for unwanted primer interactions using the Oligo Analyser Tool from IDT [24].

Four novel TaqMan primers sets were designed using the Primer3 [25] tool, ensuring that all designs would be fully specific for all of the available *Ehrlichia canis* citrate synthase sequence accessions (AY647155, CP000107, AF304143, AY615901 and JN391409) retrieved from GenBank [26]. The functionality of designs was compared using the Lightcycler Probes Master Mix (Roche Diagnostics Ltd., Burgess Hill, UK). The annealing temperatures and extension time of each primer set were optimised and the best taken forward for the evaluation (Fig. 2).

### DNA extraction and amplification conditions

DNA from 100 μl of whole blood or cultured bacteria was extracted using the DNeasy Blood and Tissue Kit (Qiagen, Hilden, Germany) according to the manufacturer’s recommendations [27].

DNA was detected by TaqMan real-time PCR using HPLC grade primers and probes (synthesised by Eurofins Genomics, Ebersberg, Germany) which were designed to target a 146 bp segment of the citrate synthase gene (gltA-For: 5′-TAG CAA CTT TAT GGG GGC CA-3′; gltA-Rev: 5′-TGA CCA AAA CCC ATT AGC CTC-3′ and the probe gltA-Probe FAM-5′-AGT AAC GTA AAG CAG TTT ATT CAA-BHQ1-3′). Each 25 μl reaction consisted of 12.5 μl 2× Lightcycler Probes Master Mix (Roche), a final concentration of 400 nM of each primer and probe and 5 μl of extracted DNA. Amplification was carried out on a Roche Lightcycler 96 System. The thermal cycle was initiated with a denaturing step for 5 min at 95 °C followed by 40 cycles 95 °C for 15 s, 56 °C for 1 min. Data was acquired in the FAM channel during the extension step of each reaction.

PCRun® reaction pellets were dissolved in 15 μl PCRun® Buffer prior to the addition of 5 μl extracted DNA. Amplification was performed using both TaqMan and PCRun® on aliquots of the same DNA sample. Each batch of 14 samples was tested against a known positive and a naïve DNA (negative control). Amplification took place for 60 min at 60 °C on a PCRun® Reader that also scored positivity in real time. In addition, all PCRun® reaction amplicons were also analysed by lateral flow at endpoint employing USTAR Disposable Nucleic Acid Detection Device (Ustar Biotechnologies Ltd, Hanzhou China [28]).

### Amplification efficiency, analytical sensitivity and specificity

The PCRun® and TaqMan assays were challenged with a 10-fold dilution series of cultured *E. canis* genome (diluted 1, 10, 100, 1000 and 10,000×). Three replicates were evaluated per copy number and test. The sensitivity achieved by each amplification was scored and the amplification efficiency for TaqMan was calculated from logarithmic regression analysis of the respective data [29].

The specificity of both PCRun® and the *gltA* TaqMan assays was challenged with DNA extracted from verified EDTA treated whole blood samples that contained an *Ehrlichia* species known to cause ehrlichiosis in dogs (*E. canis*, *E. ewingii* and *E. chaffeensis*) or the closely related species of *Anaplasma* (*A. platys* and *A. phagocytophilum*). The newly identified *E. minasensis* was also assessed via both technologies.

### Clinical sensitivity and specificity

Two methods of DNA amplification were evaluated on a collection of 33 retrospective samples consisting of known *Ehrlichia*-positive and -negative dog whole blood samples provided by Dr Tristan Cogan at the School of Veterinary Sciences (Bristol University, UK) and a further 215 prospective whole blood samples collected from dogs in Israel displaying anorexia, elevated temperature and thrombocytopaenia along with a history of recent exposure to ticks. The DNA extractions and amplifications were performed as described. The Cq-values generated using the newly developed *gltA* TaqMan method were compared with time-to-positive readings on the PCRun® Reader, and end-point analysis by USTAR Disposable Nucleic Acid Detection Device. Test comparisons were then performed on the positivity of data using MedCalc Software [30].

## Results

### Amplification efficiency, sensitivity and specificity

**Fig. 3:**
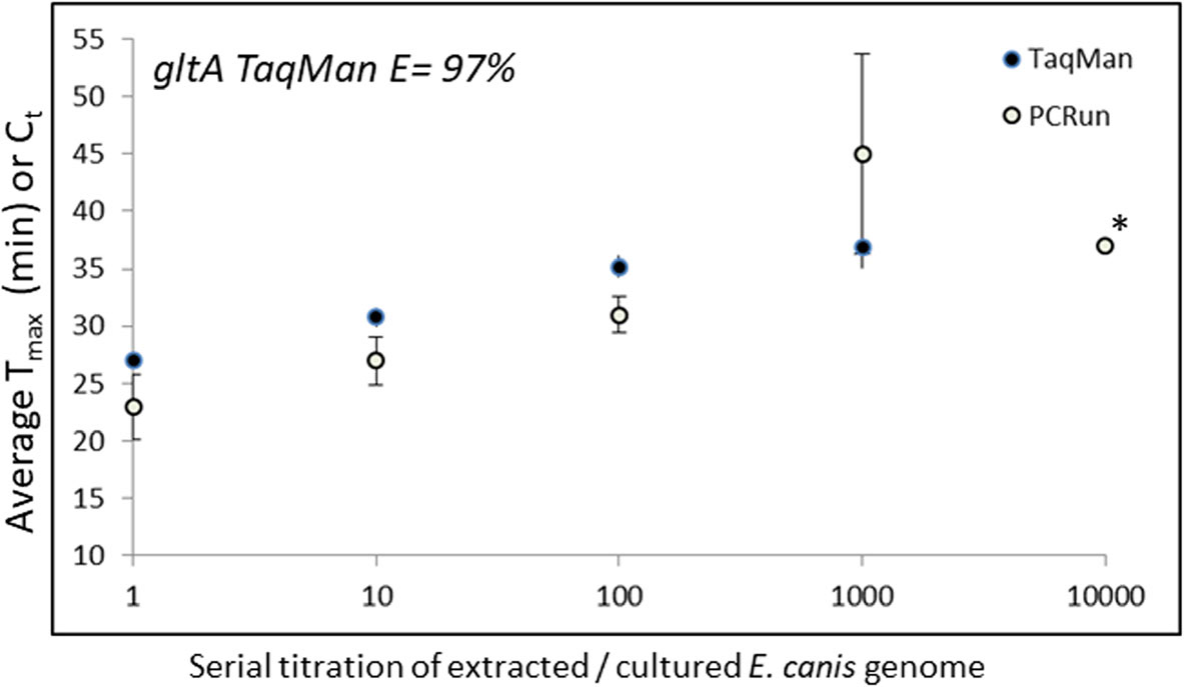
The regression analysis for PCRun® and TaqMan amplification from 10-fold serial titrations of cultured *Ehrlichia canis* genome extracts. The mean C_t_ (TaqMan) or time to maximum light output (PCRun®) was plotted against the known dilutions and the error bars show the standard deviation around this value. All replicates amplified, except for those marked with an asterisk indicating a single replicate produced a positive signal

The best performing TaqMan primer set was assessed for amplification efficiency using 10-fold serial titrations of *Ehrlichia canis* genome extracted from culture and diluted in naïve canine DNA. The calculated efficiency of this TaqMan assay was well within tolerance (*E* = 97%, Fig. 3). Furthermore, the amplification was demonstrated to have an analytical limit of detection equivalent to the commercial isothermal amplification method used as a benchmark for this work (Fig. 3). The PCR amplified product generated was analysed by gel electrophoresis and only one specific product was observed that migrated at its predicted molecular weight (150 bp; data not shown). The TaqMan and PCRun® amplification time of detection was very reproducible at all titrations of genome tested, and both assays exhibited similar kinetics. Together this data demonstrates that the *gltA* TaqMan and *16S* PCRun® amplified the various copy numbers of *E. canis* genome at acceptable speeds and with the same sensitivities (Fig. 3).

The specificity of each test was assessed with respect to the genomic DNA extracted from a number of *Ehrlichia* and closely related *Rickettsia* (Table 2). This work demonstrated that while the TaqMan PCR was highly specific for *E. canis* and *E. minasensis*, the PCRun® method could also detect all of the *Ehrlichia* and related *Anaplasma* derived *16S* sequences (Table 2).

**Table 2:** Inclusivity and exclusivity of PCRun® *Ehrlichia* molecular detection kit and gltA TaqMan PCR

Species	Sample type	Potential for amplification	
		PCRun (*16S*)	TaqMan (*gltA*)
*Ehrlichia canis*	Whole blood/culture	Yes	Yes
*Ehrlichia minasensis*	Cultured	Yes	Yes
*Ehrlichia chaffenesis*	Cultured	Yes	No
*Ehrlichia ewingii*	Whole blood	Yes	No
*Anaplasma platys*	Whole blood	Yes	No
*Anaplasma phagocytophilum*	Whole blood	Yes	No

### Clinical sensitivity and specificity

The *gltA* TaqMan assay was evaluated in a double-blind study using the *16S* ribosomal PCRun® DNA amplification as a molecular diagnostic benchmark. Both assays were challenged using 33 canine whole blood samples sourced from UK clinics. The data presented in Table 3 shows that the PCRun® and TaqMan assay deemed the same samples to be positive and negative. Further to this analysis, a more comprehensive evaluation was performed on 215 canine whole blood samples collected in Israel. The status of these samples was determined using both amplification technologies. Once more, the TaqMan assay achieved absolute sensitivity and specificity (100%) with respect to the PCRun® benchmark; whether scored by real time or endpoint analysis (Table 3, Additional file 2: Table S1 and Additional file 3: Table S2). The level of prevalence was shown to be far lower within the larger sample set tested and this is discussed.

**Table 3:** Comparison of PCRun® Canine *Ehrlichia* Kit to the *E. canis gltA* TaqMan PCR. Totals of 33 samples from the UK and 215 samples from Israel were assessed in a double-blind study, with statistical analysis performed using MedCalc Software [30] (see Additional file 2: Table S1 and Additional file 3: Table S2 for positivity data)

	UK	Israel
Benchmark	PCRun (*16S*)	PCRun (*16S*)
Test assay	Taqman (*gltA*)	Taqman (*gltA*)
Total tests	33	215
Sensitivity (%)	100	100
Specificity (%)	100	100
Accuracy (%)	100	100
Positive predictive value (%)	100	100
Negative predictive value (%)	100	100
Prevalence (%)	42	21
False discovery rate	0	0
False positive rate	0	0
False omission rate	0	0
False negative rate	0	0
Positive likelihood ratio	100	100
Negative likelihood ratio	0	0

## Discussion

This work demonstrates that both diagnostic tools have similar sensitivities with respect to the field samples studied here and this was despite differing target sequences and geographical locations. The prevalence of ehrlichiosis in suspected dogs was concordant with respect to the amplification and geographical location, demonstrating conservation of the respective targets and primer sequences within the populations of dogs tested. The specificity of each test with respect to primer interactions was also very reliable, as no false positives were called throughout the evaluation of either test.

It is also interesting to note that both of the tests investigated in this report amplify *Ehrlichia minasensis*. This is a significant result that may have clinical relevance in the future. This newly identified *Ehrlichia* species was shown to be closely related to *E. canis* based on phylogenetic analysis (Fig. 1). Furthermore, *Ehrlichia minasensis* can be readily cultured in canid cells [2]. To date little is known about the pathogenesis of this new *Ehrlichia* variant, although many *Ehrlichia* spp*.* that afflict dogs are now emerging as significant for human disease [31, 32].

The TaqMan method was shown to be more specific for canine ehrlichiosis compared to the PCRun® (Table 2), which may have applicability. It is often important to diagnose ehrlichiosis and causal species of disease early, as dogs who are not treated during the acute phase will enter into a chronic state of infection [8, 33]. Furthermore, canines with a history of chronic *E. canis* infection are hypothetically at higher risk of developing a debilitating autoimmune-mediated thrombocytopenia [34]. The clinician must therefore diagnose the species in order to define and pursue a suitable treatment protocol and lifetime follow-up of the animal. The prognosis for animals that are not diagnosed correctly is very poor. While this work demonstrates that the TaqMan method may be species-specific, the PCRun® will have a broader appeal as a diagnostic tool for emerging ehrlichiosis in dogs and humans as it is well suited towards near point-of-care.

Both assays are equally suited as diagnostic tools. Although the TaqMan PCR is approximately 20 minutes faster to complete amplification, the PCRun® reader produces real time results without requiring any data processing. Confirmation of a result can also be much more readily achieved without the risk of contamination with PCRun® assay using the USTAR device. The PCRun® system is limited to 16 simultaneous amplifications, which is clearly more suited towards clinics, and the lyophilised format of the PCRun® assay reduces facility and personnel requirements. Alternatively, the TaqMan assay could be further developed to become a quantitative PCR, which might have diagnostic or therapeutic benefits in identifying acute and chronic disease or monitoring treatment.

The prevalence of *E. canis* in both sample sets ranged from between 21–42%. The Israeli sample set represents a large group of animals exhibiting thrombocytopenia and not necessarily ehrlichiosis and this is reflected by the lower *E. canis* prevalence, while the samples obtained from Bristol University were suspect for ehrlichiosis. It was clear from the TaqMan analysis that all of the samples called positive by both techniques were either *E*. *canis* or *E*. *minasensis*. Four of the negative samples were confirmed to be positive for *Anaplasma platys* showing that this infection does not compromise the TaqMan disease status (data not shown).

## Conclusions

Both TaqMan and PCRun® methods were equally suited to diagnosing canine ehrlichiosis, achieving similar sensitivities and prevalences despite disparate amplification methods and target sequences. A newly identified species *E. minasensis* was detectable by both methods; however, closely related *A. platys* did not cross-react with TaqMan. Accurate real time diagnosis of ehrlichiosis species could help to improve prognosis for canines and humans presenting with thrombocytopenia.

## Additional files

Additional file 1: Available *Ehrlichia* sp. genome accessions. A list of the Genbank accession numbers used to generate the cladogram in Fig. 1. (XLSX 13 kb)

Additional file 2: **Table S1.** Comparison of PCRun® canine *Ehrlichia* positivity data to the *E*. *canis* gltA TaqMan PCR. Both PCRun® and TaqMan PCR assays assessed 33 genomic DNA extracts isolated from whole canine blood samples at Langford House at the University of Bristol. (XLSX 8 kb)

Additional file 3: **Table S2.** Comparison of PCRun® canine *Ehrlichia* sp. real time positivity data to the *E. canis* gltA TaqMan PCR. A total of 215 whole canine blood samples from two districts in Israel (Be’er Sheva and Kiryat Shmona) were used to evaluate both PCRun® and TaqMan PCR. (DOCX 13 kb)

## Abbreviations

*16S* rRNA: *16S* ribosomal gene
DNA: Deoxyribose nucleic acid
*dsb*: Disulfide oxidoreductase gene
*gltA*: Citrate synthase gene
IFA: Indirect fluorescent-antibody assay
IVD: *in vitro* diagnostics
PCR: Polymerase chain reaction
