# Supplementary material for: A new TaqMan method for the reliable diagnosis of Ehrlichia spp. in canine whole blood
Source: Parasit Vectors. 2018 Jun 18;11:350. doi: 10.1186/s13071-018-2914-5 (PMC6006785; doi:10.1186/s13071-018-2914-5)
Supplement: Supplementary file 3 — Table S2. Comparison of PCRun® canine Ehrlichia sp. real time positivity data to the E. canis gltA TaqMan PCR. A total of 215 whole canine blood samples from two districts in Israel (Be’er Sheva and Kiryat Shmona) were used to evaluate both PCRun® and TaqMan PCR. (DOCX 13 kb) [file 13071_2018_2914_MOESM3_ESM.docx]

**Ribosomal DNA (16s) encoding *Ehrlichia* sp accession numbers**

***Ehrlichia canis***

|AB723707|B723708|AB723709|AB723710|AB723711|AB723712|AF156785|AF156786|AF162860|AF373612|AF536827|AY621071|CP000107|DQ915970|ECU26740|ECU96437|EF011110|EF011111|EF139458|EF195134|EF195135|EU106856|EU263991|EU43994|GU810149|JF429693|JX861392|JX893522|KC479022|KC479023|KJ513194|KJ513196|KJ513197|KJ659037|M73226|NR_074283|NR_118741|EU491504|

***Ehrlichia chaffeensis***

|AF147752|AF416764|CP000236|CP007473|CP007475|CP007476|CP00747|ECU60476|NR_037059|NR_074500|

***Ehrlichia ewingii***

|EEU96436|NR_044747|

***Ehrlichia mineirensis***

|CP007474|

***Ehrlichia muris***

|AB013008|AB013009|AB196302|CP006917|NR_025962|NR_121714|GU358691|

***Ehrlichia ruminantium***

|CR767821|CR925677|CR925678|DQ647615|DQ647616|NR_074155|NR_074513|

**Citrate synthase (gltA) encoding *Ehrlichia* sp accession numbers**

***Ehrlichia canis***

|AY647155|CP000107|AF304143|AY615901|JN391409|

***Ehrlichia mineirensis***

|NZ_CP007474|

***Ehrlichia ovina***

|KP719095|

***Ehrlichia muris***

|CP006917|AF304144|HQ660497|HQ660495|HQ660496|HQ660494|

***Ehrlichia chaffeensis***

|CP007478|CP007480|CP007473|CP007479|CP007475|CP007477|CP007476|CP000236|AF304142|

***Ehrlichia ruminantium***

|CR925678|CR925677|CR767821|DQ513394|DQ513396|DQ513393|DQ513390|DQ513395|DQ513391|DQ513397|DQ513392|DQ365879|

**Disulphide oxidoreductase (DSB) encoding *Ehrlichia* sp accession numbers**

***Ehrlichia canis***

|CP000107|AF403710|DQ124260|AY236485|DQ124259|DQ124258|DQ124257|DQ124256|DQ124255|DQ124254|DQ460716|DQ460715|DQ902687|GU586135|JQ419757|

***Ehrlichia minasensis***

|JX629808|

***Ehrlichia chaffeensis***

|CP000236|AF403711|AY236484|EF375886|JQ085942|DQ902686|EF375887|F375885|

***Ehrlichia ruminantium***

|CR925678|CR767821|AF308669|CR925677|EU91925|EU919249|

***Ehrlichia muris***

|EU919248|

***Ehrlichia ewingii***

|AY428950|DQ902688|DQ151999|

**Major antigenic protein (MAP1 / p28) encoding *Ehrlichia* sp accession numbers**

**Ehrlichia muris**

|AF165814|AB178804|AF165813|AB178806|AB178807|AB178805|

**Ehrlichia ewingii**

|AF287963|AF287961|AF287966|AF287962|AF287964|

**Ehrlichia chaffeensis**

|AF393395|AF393393|AY117397|AF077734|AF077735|AF393389|AF393394|AF393391|AF077732|AF393390| |AF393388|AY117396|AF077733|F393392|

**Ehrlichia canis**

EU439942|EF014897|AF165815|GU951532|DQ460713|Y872188|F082750|AF082749|F082748|F082746|F082747| F062762|F165816|AF355200|B218278|F627981|

**Ehrlichia ruminantium**

AF368013|X74250|AF325176|EF627982|F627983|U50834|AF368001|JX477671|AF368003|AF368004|JX486797| JX486788|JX477666|DQ333230|AF368014|AF368012|AF368007|AF368005|AF368000|U50830|AF355201|CRU49843|JX486794|JX486790|JX477668||JX477670|JX477664.1|JX477665|AF355202|AY028378||JX486796||JX477672||HQ259913|HQ259911|EF627980|AF355203|AF368015|AF368011||U50833.1|EF627985|U50831|AY872189|AF368008|AF368010|JX486791.1|HQ259910|JX486799|JX486795|JX486798|JX486793|JX486792|JX477669|JX477663|JX477667|EF627984|EU272373|EU272367|HQ259912||EU272366.1|

**Ehrlichia unknown species**

|EU272363|EU272362|EU272361|EU272356|EU272354|EU272350|EU272349|EU272345|EU272344|EU272339|DQ324368|EU272360|EU272355|EU272353|EU272346|EU272342|EU272341|EU272358|EU272359|AB178802|AB178800|AB178801|AB178799|AB178794|AB178798|AB178797|AB178795|AB178796|AB178803|EU272357|EU272347.1|JN217097|JQ697886|FJ824084|FJ824083|FJ824085|EU272369|EU272351|EU272348|JQ69788|EU272371| |EU272368|
